# Neurogenesis in the neonatal rat hippocampus is regulated by sexually dimorphic epigenetic modifiers

**DOI:** 10.1186/s13293-022-00418-2

**Published:** 2022-03-07

**Authors:** S. L. Stockman, K. E. Kight, J. M. Bowers, M. M. McCarthy

**Affiliations:** 1 Department of Medicine, University of Pittsburgh Medical Center, Pittsburgh, PA 15213 USA; 2 Department of Pharmacology, University of Maryland School of Medicine, 685 W. Baltimore Street, Baltimore, MD 21201 USA; 3 School of Neuroscience, Virginia Polytechnic Institute and State University, Blacksburg, VA 24061 USA; 4 University of Maryland Baltimore, Program in Neuroscience, Baltimore, MD 21201 USA

**Keywords:** Neurogenesis, Hippocampal formation, Sex differences, Epigenetics, Development

## Abstract

**Background:**

Neurogenesis in the hippocampus endures across the lifespan but is particularly prolific during the first postnatal week in the developing rodent brain. The majority of new born neurons are in the dentate gyrus (DG). The number of new neurons born during the first postnatal week in the DG of male rat pups is about double the number in females. In other systems, the rate of cell proliferation is controlled by epigenetic modifications in stem cells. We, therefore, explored the potential impact of DNA methylation and histone acetylation on cell genesis in the developing DG of male and female rats.

**Methods:**

Cell genesis was assessed by quantification of BrdU + cells in the DG of neonatal rats following injections on multiple days. Methylation and acetylation were manipulated pharmacologically by injection of well vetted drugs. DNA methylation, histone acetylation and associated enzyme activity were measured using commercially available colorimetric assays. mRNA was quantified by PCR. Multiple group comparisons were made by one- or two-way ANOVA followed by post-hoc tests controlling for multiple comparisons. Two groups were compared by *t* test.

**Results:**

We found higher levels of DNA methylation in male DG and treatment with the DNA methylating enzyme inhibitor zebularine reduced the methylation and correspondingly reduced cell genesis. The same treatment had no impact on either measure in females. By contrast, treatment with a histone deacetylase inhibitor, trichostatin-A, increased histone acetylation in the DG of both sexes but increased cell genesis only in females. Females had higher baseline histone deacetylase activity and greater inhibition in response to trichostatin-A treatment. The mRNA levels of the proproliferative gene brain-derived neurotrophic factor were greater in males and reduced by inhibiting both DNA methylation and histone deacetylation only in males.

**Conclusions:**

These data reveal a sexually dimorphic epigenetically based regulation of neurogenesis in the DG but the mechanisms establishing the distinct regulation involving DNA methylation in males and histone acetylation in females is unknown.

## Highlights

- Neurogenesis in the dentate gyrus peaks in the early postnatal period and in the laboratory rat is significantly greater in males than females.
- Here we report divergent regulation of cell genesis in the neonatal dentate gyrus. DNA methylation is a critical regulator of the higher rates of proliferation in males. Conversely, histone acetylation is essential for cell genesis in females.

## Background

The brain is unique in being one of the first organs to develop but the last to mature and is specifically designed to modify its development in response to both internal physiological cues and external stimuli. A foundational component of brain development is the production of excess numbers of cells and synapses, which are then removed via orchestrated apoptosis, phagocytosis and pruning that integrates internal as well as environmental cues. One of the most potent physiological cues directing brain development are steroid hormones, which diverge in males and females during a critical period that begins in fetal life and extends briefly into the postnatal period in rodents [1]. However, in contrast to the common “rule” of removing cells and synapses, steroid hormones promote the formation of new synapses and in some cases new cells [2–5]. The mechanisms by which these fundamental aspects of brain development are modified by gonadal steroids are still being elucidated and appear to differ by region and endpoint, creating a complex mosaic of discrete nodes of sexually differentiated neural architecture [6]. Moreover, the contribution of genes on the X and Y chromosome to establishing and maintaining sex differences in brain and behavior is an established fact but many details remain to be elucidated [7–10].

We have previously documented a sex difference in cell genesis within the hippocampus during the very early postnatal period. Newborn male rats generate significantly more new cells in subregions of the hippocampus including CA1, CA3 and the dentate gyrus (DG), compared to female littermates [4, 11]. Treatment of newborn females with either androgens or estrogens increases proliferation in the hippocampus to the level normally found in males, while antagonists of the estrogen (ER) and androgen receptor (AR), or genetic mutation of the AR, reduce proliferation rates in males to that of females [4, 11]. Steroid receptors are nuclear transcription factors that associate with large transcriptional complexes that include histone modifying enzymes [12], and the genes coding for AR and ER are themselves subject to epigenetic regulation in the brain [13, 14]. Thus there is reciprocal cross-talk between steroid hormone receptors and the epigenome capable of regulating epochs of hormone sensitivity as well as enduring consequences of steroid exposure that may last a lifetime.

Maintenance of a cell’s proliferative state requires suppression of anti-proliferative factors in favor of expression of pro-proliferative genes. The three major cell types of the brain, neurons, astrocytes and oligodendrocytes all originate from a common stem cell precursor. The ultimate fate of a particular cell is determined by intrinsic epigenetic programming that interacts with transcription factors and environmental cues [15]. Canonical modes of epigenetic regulation include DNA methylation of cytosine residues proximal to guanines (CpGs) and acetylation of histone tails. DNA methylation traditionally represses transcription [16, 17], whereas acetylation of histones stimulates transcription [18, 19]. The addition and removal of these epigenetic modifiers is coordinated by unique enzymes. Histone acetyltransferase (HAT) enzymes catalyze the addition of acetyl moieties and in the opposing reaction, histone deacetylase (HDAC) enzymes remove them. DNA methyltransferases catalyze the transfer of a methyl group from S-adenosyl-L methionine to cytosines to produce methylated DNA. The reverse reaction is significantly more disputed and complicated, but a generally excepted mechanism involves a multi-step enzymatic action, whereby 5mC is hydroxylated by a family of TET (Ten–Eleven-Translocation) enzymes to form 5hmC, which is deaminated to convert 5hmC to 5-hydroxymethyl-uracil. This results in a DNA mismatch that stimulates the base excision repair (BER) pathway to correct the miss paired bases [20]. GADD45 (Growth Arrest and DNA Damage) enzymes facilitate active DNA demethylation through the recruitment of thymine–DNA glycosylase and other repair enzymes to the site of demethylation [21].

Given the importance of epigenetic coordination in the regulation of stem cell status and determination of cell fate, we hypothesized that elevated cell genesis in the male neonatal DG is due to sexually differentiated epigenetic regulation within the DG and so we explored the role of both histone and DNA modifications. We found opposing roles for each in males versus females, with an impact of DNA methylation on cell genesis in males, while histone modifications altered the cell genesis profile of females.

## Methods

### Animals

All animal experiments were conducted with approval from the University of Maryland School of Medicine Institutional Animal Care and Use Committee and performed in accordance with national animal care and use guidelines. Adult Sprague Dawley rats (Harlan) were mated in our facility. Mating was confirmed by the presence of sperm in vaginal smears. Pregnant females were isolated and allowed to deliver normally. Cages were checked daily for the presence of pups to determine the timing of birth. Pups were sexed and treated within 6 h of detection in the nest. All animals were provided ad libitum food and water and were maintained on a reverse 12 h light/dark cycle.

### Drug administration

Drugs with known epigenetic modification effects were administered on the animals’ day of birth (denoted as postnatal day 0—PN0) and PN1. Trichostatin A (TSA) is a histone deacetylase inhibitor and was administered intraperitoneally (IP) at a dose of 0.5 mg/Kg in 5% DMSO in saline, and the injection site was sealed with Vetbond tissue adhesive (3 M). Control animals received the same volume and route of 5% DMSO in saline. Zebularine (ZEB) is a DNA–methyl-transferase (DNMT) inhibitor and was injected intracerebroventricularly (ICV) at a dose of 300 ng in 1% DMSO in saline. Control animals received the same volume and route of 1% DMSO in saline. Bilateral ICV injections were performed under bright light illumination, allowing for visualization of the cranial landmark, Bregma, to approximate the location of the lateral ventricles. Injections were targeted at 1 mm rostral and 1 mm lateral to Bregma, to avoid penetration of the hippocampus. A 23-gauge, 1 μl Hamilton syringe was lowered 2 mm below the surface of the skull to reach the ventricle. Each hemisphere was infused with a 1ul volume over 60 s. Prior to ICV injections, pups were cryoanesthetized for approximately 10 min. Following drug treatments, animals received small subcutaneous injections of ink in the paw for treatment group identification.

### Tissue dissection

To dissect the DG, brains were bisected on the sagittal plane in ice-cold phosphate-buffered saline (PBS; pH 7.4), and thalamic tissue was removed to expose the ventricular surface of the hippocampus. The entire DG was removed as a discrete structure by inserting a fine gauge needle along the length of the hippocampal fissure. Fiber projections along the dentate axis proximal to CA3 were removed with fine forceps. DG were placed in fresh microcentrifuge tubes, immediately frozen on dry ice and stored at − 80 °C until use.

### Protein extraction

DG samples were homogenized in 75 μL radioimmunoprecipitation assay buffer (RIPA buffer) with phosphatase and protease inhibitors both at 1:1000 concentration. Samples were centrifuged at 4000 rpm for 10 min and the supernatant collected and transferred to a fresh tube. Protein concentration was determined by Bradford assay. Aliquots were made of the nuclear fraction and used immediately or frozen at − 80 °C until use in the HAT activity assay.

### Nuclear extraction

Nuclear extracts were generated from DG samples using the EpiQuick Nuclear Extraction Kit (Epigentek) following the manufacturer’s protocol. Each mg of tissue was homogenized in 5 μL cold pre-extraction buffer containing dithiothreitol (DTT) diluted 1:1000 and incubated on ice for 15 min before centrifugation for 10 min at 12,000 rpm at 4 °C. The supernatant was removed and nuclear pellets were suspended in ice cold extraction buffer containing DTT and protease inhibitor diluted 1:1000 and incubated for 15 min on ice with vortexing every 3 min. The suspensions were sonicated three times for 10 s and then centrifuged for 10 min at 14,000 rpm at 4 °C. The supernatant containing the nuclear extracts was transferred to a fresh tube. Protein concentration was determined by Bradford assay. Aliquots were made of the nuclear extract and used immediately or frozen at − 80 °C until use in the DNA methyltransferase or HDAC activity assays.

### Histone extraction

Histones were extracted from DG samples with the EpiQuick Total Histone Extraction Kit (Epigentek). Pre-lysis buffer was added to the tissue at a ratio of 1 ml/200 mg of tissue and tissues pieces were disaggregated with a Dounce homogenizer. The solution was centrifuged at 3,000 rpm for 5 min at 4 °C and the supernatant removed before 1 μl/mg of lysis buffer was added to the pellet and samples were incubated at 4 °C overnight. Samples were centrifuged at 12,000 rpm for 5 min at 4 °C and the supernatant fraction containing the acid-soluble proteins transferred to a new vial. DTT was added to the balance buffer at 1:500 ratio and 0.3 μl of balance-DTT buffer was added to every 1 μl of supernatant. Protein concentration was determined by Bradford assay. Aliquots were made, frozen at − 80 °C and utilized for western immunoblotting.

### DNA isolation

DNA from DG samples was extracted using the Wizard Genomic Purification Kit (Promega) following the manufacturer’s instructions. Briefly, tissue was homogenized in the Nuclei Lysis Solution and incubated at 65 °C for 30 min. Proteinase K was added to each sample and incubated at 55 °C with gentle shaking overnight. Protein Precipitation Solution was added and samples vortexed vigorously and chilled on ice for 5 min. Samples were centrifuged at 16,000 *g* to remove protein, supernatant transferred to a clean microcentrifuge tube containing 600uL of isopropanol and the solution mixed by inversion until white thread-like strands of DNA formed a visible mass and then centrifuged for 1 min at 16,000 *g*. The supernatant was removed and the pellet washed in ethanol, allowed to air-dry for 15 min and rehydrated in 100 μl of DNA Rehydration Solution for 1 h at 65 °C and then DNA stored at 4 °C until use in the global DNA methylation study.

### Quantification of global DNA methylation

Males and females were administered ZEB (300 ng in 1% DMSO in saline, i.c.v) or vehicle on PN0 and PN1 and DG collected 2 h later. A second cohort of DG samples collected from untreated PN1 males and females were also assessed to confirm results. DNA was isolated from samples as described and the MethylFlash Methylated DNA Quantification Kit (Epigentek) was used following the manufacturer’s instructions and 100 ng of sample DNA was bound to plate wells and probed with an anti-5-methylcytosine antibody and HRP-linked secondary to produce a colorimetric reaction read at an absorbance of 450 nm using a microplate reader. Percent methylation was calculated using the following equation supplied by the manufacturer:

$$5-\mathrm{mc}\%=\frac{\mathrm{Sample OD}-\mathrm{Negative Control}}{\mathrm{Slope of standard curve} \times S}\times 100\%$$

where *S* is the amount of input sample DNA in ng.

### Immunohistochemical quantification of BRDU

Pups were injected with bromodeoxyuridine (BrdU, Sigma Aldrich), a synthetic thymidine analog used to detect proliferating cells, 2 h after drug treatment on PN0–1 at a dose of 100 mg/kg s.c. Six hours after BrdU administration, pups were transcardially perfused with 0.9% saline and 4% paraformaldehyde. Brains were post-fixed for 48 h and allowed to sink in 30% sucrose prior to cryosectioning throughout the rostrocaudal extent of the hippocampus. Sections were mounted to slides and heated in 0.1 M citric acid (pH 6.0), rinsed in PBS, incubated in trypsin for 10 min, denatured in 2 M HCl:PBS for 30 min, rinsed and incubated with mouse antibodies to BrdU (BD Biosciences diluted 1:500 in 0.5% Tween-20). The next day, slides were rinsed, incubated with biotinylated anti-mouse (1:200, Vector) for 60 min, rinsed, incubated with avidin–biotin complex (1:500; Vector), rinsed and reacted in 0.01% DAB. Slides were counterstained with cresyl violet, dehydrated, cleared and coverslipped. Unbiased stereology was used to estimate the number of BrdU + cells in the hippocampus using the optical dissector method (West et al. 1991). StereoInvestigator software (MBFbioscience, Williston, VT) was used to delineate the granule cell layer of the dentate gyrus (DG) in each hemisphere. Analysis of BrdU + cells was conducted on the left and right hemisphere of 4 sections of the dorsal hippocampus and an estimation of total cells was generated.

### Western immunoblotting to quantify histone acetylation

To verify that the dose of TSA used for treatment increased histone acetylation, males and females were administered TSA (0.5 mg/Kg in 5% DMSO in saline; i.p.) or vehicle on PN0 and PN1. DG were collected 2 h following the last TSA administration. A second cohort of DG collected from untreated PN1 males and females served as controls. Histone extracts form the DG were prepared at a protein concentration of 2.5 μg/20 μl and electrophoresed in separate lanes on a 4–20% SDS–polyacrylamide gel (Invitrogen) and transferred to a nitrocellulose membrane (Bio-Rad). Membranes were blocked in Odyssey Blocking Buffer (LI-COR), diluted 1:1 with tris-buffered saline (TBS) for 1 h at room temperature and then incubated overnight at 4 °C in primary antibody, anti-acetyl-histone H3 (1:5000, Millipore), anti-acetyl-histone H4 (1:2000) or histone H3 (1:1000, Cell Signaling Technologies). Following a 1 h incubation with anti-rabbit (IR800; 1:20,000) and anti-mouse (IR700; 1:20,000) IRDye-linked secondary antibodies in Odyssey Blocking Buffer (LI-COR) diluted 1:1 with 0.1% tween in TBS, the immunoreactive bands were detected using the Odyssey Clx infrared imaging system (LICOR). The protein of interest was detected as a band with a relative molecular mass of ~ 17 kDA for acetyl-histone H3, ~ 10kDA for acetyl-histone H4 and ~ 17 kDA for histone H3. Acetylated histone expression was normalized to total H3 expression.

### Analysis of epigenetic enzyme gene expression by qPCR

On PN0, DG was collected from untreated males and females. RNA was extracted from samples using the RNeasy Kit (Qiagen) DNase digestion per the manufacturer’s protocol. Single-strand complementary DNA was synthesized with a High Capacity cDNA Synthesis Kit (Applied Biosystems) by mixing 1 μg total RNA with 4 μl random hexamers, 4 μl of 10 × RT Buffer, 4 μl of 25 × dNTP Mix, 2 μl of MultiScribe reverse transcriptase and 2 μl of RNase inhibitor and bringing the total volume to 40 μl with nuclease free water. The mixture was incubated at 25 °C for 10 min, 37 °C for 2 h and 85 °C for 5 min and stored at − 20 °C until used. Aside from GAPDH, which was designed in Primer Express (version 3.0, Applied Biosystems), all other primers were designed at http://www.ncbi.nlm.nih.gov. All primers were synthesized by Integrated DNA Technologies (IDT). qPCR was quantified using the standard curve method on a ViiA 7 real-time PCR machine (Applied Biosystems) using ViiA 7 software (version 1.1). The standards were generated by pooling an equal amount of cDNA from all the samples and diluting the pool 1:20, 1:60, 1:180, 1:540, 1:1620. Values attained from the 1:60 standard were defined as one genomic equivalent (GE). The cDNA from each sample was diluted to 1:60. Performing all reactions in triplicate, 5 μl of each diluted sample or standard was then added to 15 μl of Power SYBR Green PCR Master Mix (Applied Biosystems) containing 100 nM of the primer pairs above and cycled in the real-time machine as follows: 95 °C for 10 min, followed by 40 cycles of 95 °C for 15 s and an extension step of 60 °C for 60 s. Melting curves were generated at 0.1 °C increments between 65 °C and 95 °C after the 40 cycles. Threshold fluorescence was set to a value that generated cycle thresholds from the standard curve with a regressed exponential growth of 2 (*R*^2^ > 0.98). The GE for each sample was determined against the standard curve. The GE for the gene of interest for each sample was normalized to the GE for GAPDH for each sample.

### Assessment of DNMT activity

Nuclear extracts were prepared from DG collected from PN1 untreated males and females and 2 μg of nuclear extracts from the DG analyzed in triplicate for relative levels of DNMT activity using the EpiQuik DNA Methyltransferase Activity Assay (Epigentek) according to the manufacturer’s instructions and designed to measure total DNMT activity from all three isoforms (DNMT1, DNMT3a and DNMT3b). In this assay, strip wells were coated with a cytosine-rich DNA substrate. DNMT enzymes from the nuclear extract samples transfer a methyl group to the cytosines from the methyl-donor molecule, Adomet, to methylate the DNA substrate. The methylated DNA is then recognized with an anti-5-methylcytosine antiserum. The amount of methylated DNA, which is proportionate to enzyme activity was colorimetrically quantified using 450 mm absorbance readings from a microplate reader.

### Quantification of HDAC activity

Nuclear extracts were prepared from DG collected from PN0 untreated males and females. Total relative HDAC activity was measured with the EpiQuick HDAC Activity/Inhibition Assay Kit (Epigentek) according to the manufacturer’s instructions. The kit contains an acetylated histone substrate that is stably captured on strip wells. Three micrograms of nuclear extracts were added to wells and HDACs in the sample bound to and deacetylated the substrate. The un-deacetylated substrate was recognized with an acetylated histone antibody. HDAC activity, which is inversely proportional to the enzyme activity was colorimetrically quantified using 450 mm absorbance readings from a microplate reader. HDAC activity analyzed as OD/h/ml was calculated using the following equation supplied by the manufacturer:

$$\mathrm{HDAC activity}= \frac{[\mathrm{OD }\left(\mathrm{control}-\mathrm{blank}\right)-\mathrm{OD }(\mathrm{sample}-\mathrm{blank})}{\mathrm{reaction time}} \mathrm{sample dilution}$$

To assess the effects of inhibition, 5 μl of 100 μM of TSA or vehicle was added to each well and allowed to incubate with the sample.

### Determination of histone acetyl transferase (HAT) activity

Relative HAT activity was quantified in PN1 DG with the Histone Acetyltransferase Activity Assay Kit (Abcam) according to the manufacturer’s instructions. This kit measures total HAT activity using strip wells coated with peptide substrate and a solution with the cofactor acetyl-CoA. Acetylation of the peptide substrate by functional HATs initiates release of the free form of CoA that acts as an essential coenzyme for production of NADH. NADH is then detected spectrophotometrically following reaction with a soluble tetrazolium dye. Samples of whole-cell tissue extracts (40 μl of 1 μg/μl) were incubated with assay mix at 37 °C for 1 h. The plate was assessed in a microplate reader at 440 nm. Data was analyzed as the relative O.D. value per μg.

### Statistical analysis

All data are presented as the mean ± standard error of the mean (SEM). Comparisons of proliferation, percent methylation, histone acetylation and BDNF expression with either ZEB or TSA and sex were made using either single-factor or two-way ANOVA, as appropriate, followed by post hoc pairwise comparisons with *t* tests using Bonferroni correction to control for familywise error. Sex differences in percent methylation, DNMT activity, histone acetylation, HDAC and HAT activity, as well as gene expression were evaluated by independent samples *t* test. Effect sizes were calculated as partial eta squared (*η*^2^_*p*_) for main effects from ANOVA analyses, and Cohen’s *d* for post-hoc and *t* test comparisons. *GraphPad* Prism 9.0 software (GraphPad Software) was used for statistical analysis. Statistical significance was set at *p* ≤ 0.05.

## Results

### Experiment 1A: effect of treatment with the DNMT inhibitor ZEB on cell proliferation in the neonatal DG

**Fig. 1:**
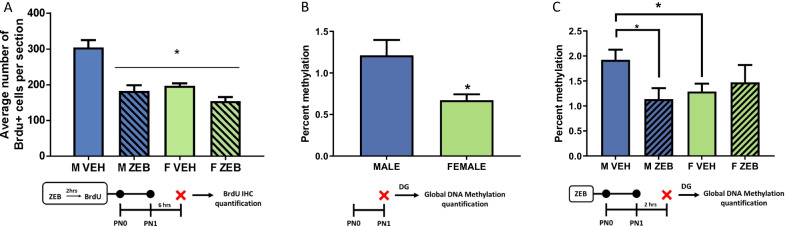
DNMT inhibitor Zebularine decreases proliferation and DNA methylation in the DG of newborn male rats, but not female rats. **A** Administration of ZEB on PN0 and PN1 interacted with sex to affect proliferation in the DG, such that males generated more new cells than females and ZEB decreased proliferation in the DG, but did not further reduce proliferation in the female DG (*p < 0.05, compared to vehicle-treated males). **B** On PN1, DNA in the DG of males is nearly twice as methylated as DNA in the female DG (*p < 0.05). **C** More DNA was methylated in the DG of males compared to females and ZEB significantly reduced DNA methylation in the DG of males but caused no further reduction in females (*p < 0.05, compared to vehicle-treated males)

Assessment of proliferation in the developing DG following ZEB administration demonstrated that treatment and sex interacted to alter cell genesis in the DG (*F*[1,13] = 15.68, *p* = 0.0016, *η*^2^_*p* = _0.5467; *n* = 4–5 rats/group from 2 L; Fig. 1A). Data confirmed the previously reported sex difference in that vehicle-treated males administered BrdU on PN0 and PN1 had more BrdU + cells in the DG granule cell layer than vehicle treated female littermates on PN2 (*t*[10] = 5.1536, *p* = 0.0004, *d* = 2.975). ZEB treatment significantly reduced proliferation in the male DG (*t*[6] = 3.991, *p* = 0.0072, *d* = 2.822), but had no effect on females (*t*[7] = 1.123, *p* = 0.2985, *d* = 0.7486), thereby eliminating the sex difference.

### Experiment 1B: Effect of treatment with the DNMT inhibitor ZEB on DNA methylation in the neonatal DG

We hypothesized that the sex-specific effect of ZEB treatment was a function of higher DNA methylation in males and tested this by assessment of global DNA methylation. There was more DNA methylation detected in cells from the DG of males compared to females (*t*[10] = 2.728, *p* = 0.0213, *d* = 1.5752; *n* = 6 rats/sex from 2 L; Fig. 1B). Administration of ZEB on PN0 and PN1 interacted with sex to affect DNA methylation differently in the DG of males and females collected 2 h after ZEB treatment on PN1 (*F*[1,22] = 4.251, *p* = 0.05, *η*^2^_*p* = _0.1619; *n* = 6–7 rats/group from 3 L; Fig. 1C). Global DNA methylation was again greater in the vehicle-treated male DG, compared to vehicle-treated females (*t*[12] = 2.462, *p* = 0.0299, *d* = 1.3146), but following ZEB administration it was significantly reduced in males (*t*[11] = 2.617, *p* = 0.0239, *d* = 1.4558), but not in females (*t*[11] = 0.5080, *p* = 0.6215, *d* = 0.2744), resulting in no difference between ZEB treated males and females.

### Experiment 2: quantification of DNA methylating enzyme mRNA and activity and demethylating enzyme mRNA in the developing DG of males and females

**Fig. 2:**
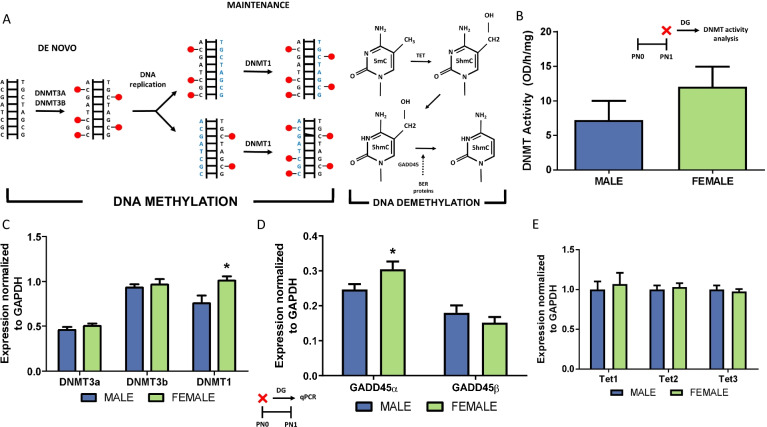
Newborn male DG expresses less of the demethylating enzyme, Gadd45α.  **A** Enzymes important for DNA methylation and demethylation. **B** Male and female DNMT activity was not significantly different in the DG at PN1. **C** Within the DG, expression of the de novo DNA methyltransferase enzymes, DNMT3a and DNMT3b did not differ at PN0. Females expressed more DNMT1 than males on PN0 in this brain region (*p < 0.05, compared to males). **D** Females expressed significantly more Gadd45α in the DG on PN1, but expression of Gadd45β did not differ between the sexes (*p < 0.05). **E** At PN0, males and females expressed similar amounts of the demethylating family of TET enzymes

We next explored the DNMT enzymes regulating de novo DNA methylation, evaluating the two isoforms, DNMT 3A and 3B, and maintenance methylation which is generally mediated by DNMT1 (Fig. 2A). Quantification of pan-DNMT activity on PN1 revealed no significant difference between males and females (*t*[18] = 1.207, *p* = 0.2429, *d* = 0.5397; *n* = 10 rats/sex from 3 L; Fig. 2B). To test for the potential of a sex differences in the amount of the three DNMTs, gene expression was assessed via qPCR. On PN0, neither expression of DNMT3a (*t*[14] = 1.045, *p* = 0.3138, *d* = 0.5132; Fig. 2C) or DNMT3b (*t*[14] = 0.4875, *p* = 0.6334, *d* = 0.2402; Fig. 2C) differed between the sexes. Unexpectedly, females expressed more DNMT1 than males on PN0 (*t*[14] = 2.567, *p* = 0.0224, *d* = 1.281; Fig. 2C). We also explored the GADD enzymes that participate in base excision repair (Fig. 2A). Gene expression of the demethylating enzyme GADD45α was significantly lower in male DG on PN0 compared to females (*t*[14] = 2.161, *p* = 0.0485, *d* = 1.068; Fig. 2D). Expression of GADD45β was not significantly different between males and females (*t*[14] = 1.052, *p* = 0.3108, *d* = 0.5237) and no sex difference was evident in expression of any of the TET family members assessed on PN0 (Tet1: *t*[14] = 0.7051, *p* = 0.3863, *d* = 0.1921; Tet2: *t*[14] = 0.4576, *p* = 0.6542, *d* = 0.2287; Tet3: *t*[14] = 0.4345, *p* = 0.6705, *d* = 0.2155; Fig. 2E). Gene expression was evaluated in *n* = 8 rats/group from 8 L.

### Experiment 3: effect of treatment with the histone deacetylase inhibitor TSA on histone acetylation and cell proliferation in the neonatal DG

**Fig. 3:**
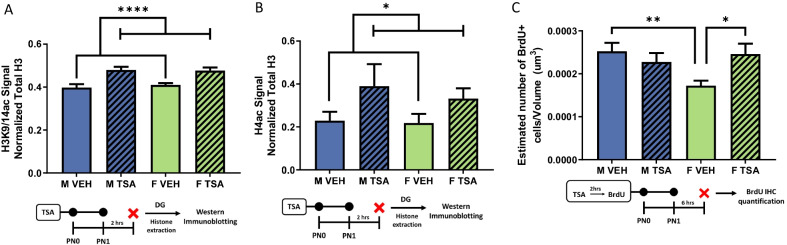
HDAC inhibitor TSA increases proliferation in the DG of females only. **A**, **B** Administration of 0.5 mg/Kg of TSA, significantly increased H3K9/14 and H4 acetylation in the DG of newborn males and females (main effect of treatment, *p < 0.05, ****p < 0.0001). **C** Treatment of TSA interacted with sex to affect proliferation in the DG as TSA treatment increased proliferation only in the female DG (significant interaction, *p < 0.05, **p < 0.01, compared to vehicle-treated females)

Inhibition of HDAC activity with TSA increased acetylation of lysines 9 and 14 on histone 3 (H3K9/14; *F*[1,18] = 27.71, *p* < 0.0001, *η*^2^_*p* = _0.6062; *n* = 5–6 rats/group from 3 L; Fig. 3A) and pan-acetylation of histone 4 (H4; *F*[1,14] = 5.1, *p* = 0.0404, *η*^2^_*p* = _0.2670; *n* = 4–5 rats/group from 3 L; Fig. 3B), equally in males and females, confirming the efficacy of the inhibitor. Treatment with TSA impacted cell proliferation in a sex-specific manner (*F*[1,28] = 6.376, *p* = 0.0175, *η*^2^_*p* = _0.1855; *n* = 7–9 rats/group from 3 L; Fig. 3C). Vehicle-treated males again had more BrdU + cells in the DG than vehicle-treated females on PN1 (*t*[15] = 3.389, *p* = 0.0040, *d* = 14.486), but TSA treatment of females increased the number of BrdU + cells to male-like levels (*t*[13] = 2.836, *p* = 0.0140, *d* = 15.390). There was no change in BrdU + cell number in males treated with TSA (*t*[15] = 0.8753, *p* = 0.3952, *d* = 0.4273.

### Experiment 4: quantification of histone deacetylation enzymes (HDAC) in the DG of males and females

**Fig. 4:**
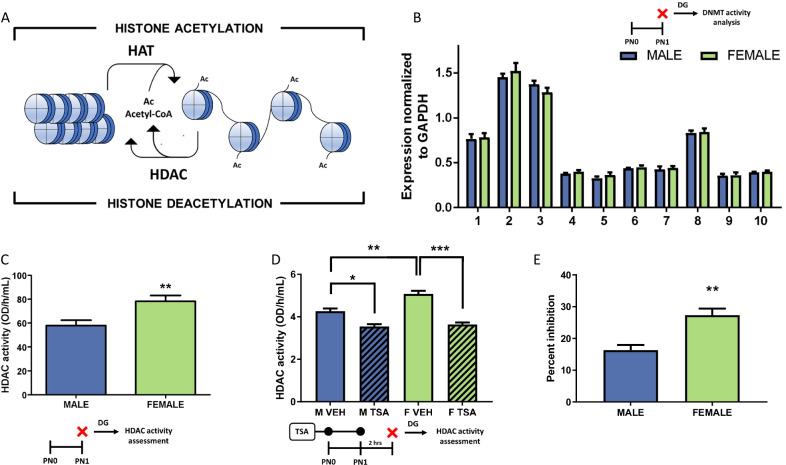
HDAC activity is greater in the newborn female DG and the degree of inhibition of HDAC activity by TSA is greater in females.  **A** Enzymes important for histone acetylation and deacetylation. **B** At PN0, expression of Class I, Class IIa and IIb HDACs quantified by qPCR did not differ by sex in the DG. **C** On PN1 HDAC activity was greater in the female DG (**p < 0.01). **D** In a separate cohort of animals, the sex difference in HDAC activity was confirmed (**p < 0.01, compared to vehicle treated males). While TSA significantly reduced HDAC activity in the DG of both sexes, there was a more robust decrease in HDAC activity within the female DG (*p < 0.05, effect of TSA treatment in males; ***p < 0.001, effect of TSA treatment in females). **E** TSA induced a greater percentage of inhibition of HDAC activity in the DG of females compared to males (**p < 0.01)

The balance of histone acetylation is achieved by opposing actions of HATs, enzymes responsible for histone acetylation, and HDAC enzymes, which coordinate deacetylation (Fig. 4A). There are 10 different isoforms of HDAC in rats, and each was quantified by qPCR. In the developing DG, males and females expressed similar quantities of all 10 HDAC enzymes targeted by TSA (Fig. 4B; *n* = 8 rats/sex from 8 L) including Hdac1 (*t*[14] = 0.2524, *p* = 0.8044, *d* = 0.4664), Hdac2 (*t*[14] = 0.6984, *p* = 0.4964, *d* = 0.3469), Hdac3 (*t*[14] = 0.1.418, *p* = 0.1780, *d* = 0.7115), Hdac4 (*t*[14] = 1.141, *p* = 0.2729, *d* = 0.5683), Hdac5 (*t*[14] = 1.0300, *p* = 0.3203, *d* = 0.5132), Hdac6 (*t*[14] = 0.6908, *p* = 0.5010, *d* = 0.3457; Hdac7 (*t*[14] = 0.3553, *p* = 0.7277, *d* = 0.1783), Hdac8 (*t*[14] = 0.8534, *p* = 0.1882, *d* = 0.0938), Hdac9 (*t*[14] = 0.1315 *p* = 0.8972, *d* = 0.0644) and Hdac10 (*t*[14] = 0.6164, *p* = 0.5475, *d* = 0.3292). While there were no differences in expression, on PN1 HDAC activity in the DG of females was greater than HDAC activity in the DG of males (*t*[12] = 3.057, *p* = 0.0100, *d* = 1.634; *n* = 7 rats/sex from 2 L; Fig. 4C). Quantification of HDAC activity following inhibition with TSA confirmed higher HDAC activity in females with a main effect of sex (*F*[1,12] = 8.347, *p* = 0.0136, *η*^2^_*p* = _0.4102; *n* = 8 rats/group from 2 L; Fig. 4D). TSA treatment reduced HDAC activity in both males and females (*F*[1,12] = 190.1, *p* < 0.0001, *η*^2^_*p* = _0.9406), but there was also a significant interaction between treatment and sex (*F*[1,12] = 20.80, *p* = 0.0007, *η*^2^_*p* = _0.6341) with a higher percent inhibition in females (t[12] = 4.138, *p* = 0.0014, *d* = 2.213).

### Experiment 5: Quantification of histone acetyl transferase (HAT) enzyme in the DG of males and females

**Fig. 5:**
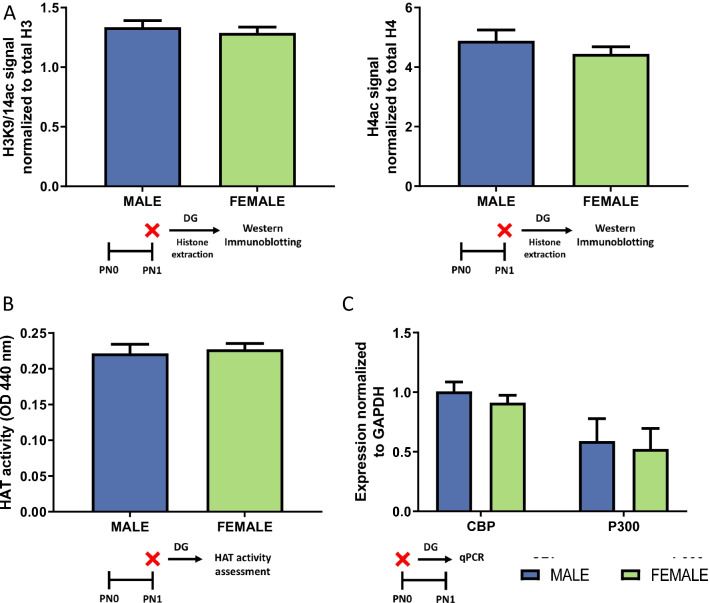
There is no sex difference in histone acetylation or HAT activity or protein. **A** On PN1, males and females had similar levels of acetylation of both H3K9/14 and H4. **B** At PN0, expression of HATs, CBP and P300, were the same in males and females. **C** There were no sex difference win HAT activity within DG on PN1

There was no sex difference in histone acetylation of H3K9/14 (*t*[18] = 0.6493, *p* = 0.5244, *d* = 0.2924), or H4 (*t*[18] = 1.016, *p* = 0.3231, *d* = 0.4543; *n* = 10 rats/sex from 3 L; Fig. 5A). In contrast to HDAC activity, there was no significant difference in HAT activity between males and females (*t*[11] = 0.3790, *p* = 0.7119, *d* = 0.0203; *n* = 6–7 rats/sex from 2 L; Fig. 5B). We also examined two HATs associated with sex differences elsewhere in the brain, CBP and P300 [22, 23]. On PN0, males and females similarly expressed these HATs (CBP *t*[14] = 0.9478, *p* = 0.3593, *d* = 0.4747 and P300 *t*[14] = 0.2615, *p* = 0.7975, *d* = 0.1308; *n* = 8 rats/sex from 8 L; Fig. 5C).

### Experiment 6: treatment with a DNMT inhibitor and HDAC inhibitor within the same cohort of animals

**Fig. 6:**
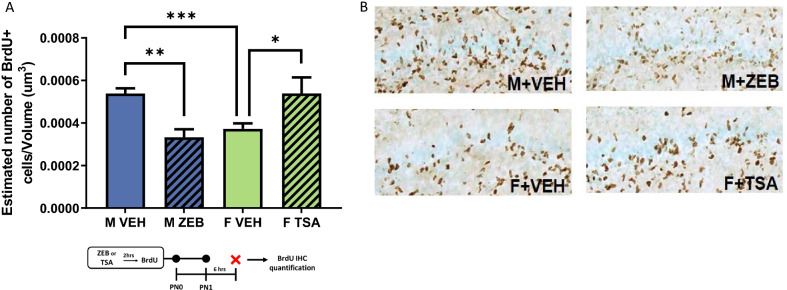
Treatment with ZEB decreases proliferation in the newborn male DG, while TSA treatment increases proliferation in female DG. **A** Treatment with ZEB and TSA within the same cohort, to eliminate potential liter effects or other bias, confirmed that ZEB administration decreases proliferation in the male DG and TSA administration increases proliferation in the female DG (***p < 0.001, sex difference in proliferation; **p < 0.001, effect of ZEB treatment in males; *p < 0.05, effect of TSA treatment in females). **B** Representative images of BrdU labeling in the DG of male rats treated with ZEB or vehicle and female rats treated with TSA or vehicle on PN0 and PN1

To confirm treatment responses were not due to litter effects or other unknown experimental biases, the effect of ZEB treatment on males and TSA treatment on females was tested within the same cohort of animals. This treatment paradigm replicated the interaction of drugs with sex evident in individual cohorts (*F*[3,20] = 6.792, *p* = 0.0024, *η*^2^_*p* = _0.5046; *n* = 6–7 rats/group from 2 L; Fig. 6A). As previously reported, vehicle-treated males had more BrdU + cells than vehicle-treated females (*t*[11] = 4.617, *p* = 0.0007, *d* = 1.295). Treatment of males with ZEB decreased BrdU + cell number compared to vehicle-treated males (*t*[10] = 4.546, *p* = 0.0011, *d* = 2.652), while treatment of females with TSA increased BrdU + cell number compared to vehicle-treated females (*t*[10] = 2.379, *p* = 0.0387, *d* = 1.032). Representative images are found in Fig. 6B.

### Experiment 7: quantification of mRNA for BDNF in males and females with and without ZEB or TSA treatment

**Fig. 7:**
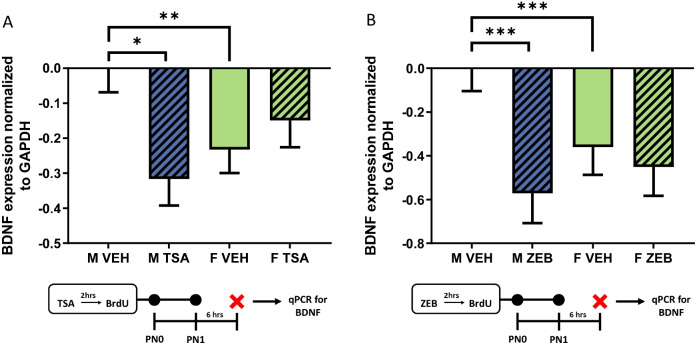
Both TSA and Zebularine treatment decrease BDNF expression but only in the male DG. **A** Consistent with previous reports, males expressed more BDNF in the DG compared to females (**p < 0.01). TSA administration decreased BDNF expression in the male DG (*p < 0.05), while expression in the female DG was unchanged with drug treatment. **B** Again, BDNF expression was greater in the male DG relative to females (***p < 0.001). ZEB treatment also reduced BDNF expression in the male DG (***p < 0.001), but had no effect on expression in the female DG

Treatment with TSA differentially altered BDNF expression in developing male and female DG (*F*[1,31] = 7.574, *p* = 0.0098, *η*^2^_*p* = _0.1963; *n* = 8–9 rats/group from 3 L; Fig. 7A). Consistent with previous data, males expressed more BDNF in the DG compared to females (*t*[16] = 3.091, *p* = 0.0070, *d* = 1.178). BDNF expression decreased in the male DG following TSA administration (*t*[15] = 2.418, *p* = 0.0288, *d* = 1.457), while expression in the female DG remained the same regardless of treatment (*t*[16] = 1.549, *p* = 0.1408, *d* = 0.3968). There was a trend for ZEB administration to interact with sex to affect BDNF expression (*F*[1,23] = 3.409, *p* = 0.0778, *η*^2^_*p* = _0.1291; *n* = 6–8 rats/group from 4 L; Fig. 7B). Again, BDNF expression was greater in the DG of males compared to females (*t*[10] = 5.373, *p* = 0.0003, *d* = 1.266). Expression of BDNF decreased in the male DG with ZEB treatment (*t*[12] = 8.523, *p* = 0.0001, *d* = 1.745), but not in the female DG (*t*[11] = 1.260, *p* = 0.2336, *d* = 0.2751). ZEB treatment eliminated the sex difference in BDNF expression (*t*[13] = 1.724, *p* = 0.1084, *d* = 0.3258).

## Discussion

The hippocampus is a critical brain region for regulating both the physiological response to stress, by mediating negative feedback control on glucocorticoid release, and to the process of forming and storing memories, particularly those that include a spatial component. These divergent and complex functions also differ in males and females in a variety of ways that depend on life stage, context and past experience [24]. Sex differences in neurophysiological parameters in the adult hippocampus abound [25, 26], including aspects of adult neurogenesis [26], but there has been relatively little attention paid to the potential for sex differences in the hippocampus as it develops. Our observation in the rat of a sex difference in neuronal proliferation in the dentate gyrus during the first postnatal week of life [4], is one example but the factors regulating differential cell genesis in males and females are poorly understood. The function of the sex difference in neuronal proliferation early in life also remains elusive, particularly in light of the fact that the overall size of the hippocampus does not vary much between males and females once mature [27]. This implies that compensatory apoptosis in males balances out the increased proliferation. However, this is not without consequences as it would mean that as they mature, the male hippocampus consists of relatively “younger” neurons compared to the female. High rates of proliferation in the hippocampus have been tied to neonatal amnesia [28], although sex differences have not been explored. Recently it was found that prior to puberty females have enhanced learning and more robust long-term potentiation compared to males, a sex difference that reverses once reproductive maturity is achieved [29]. Thus, there are multiple potential functional impacts of this sex difference.

**Fig. 8:**
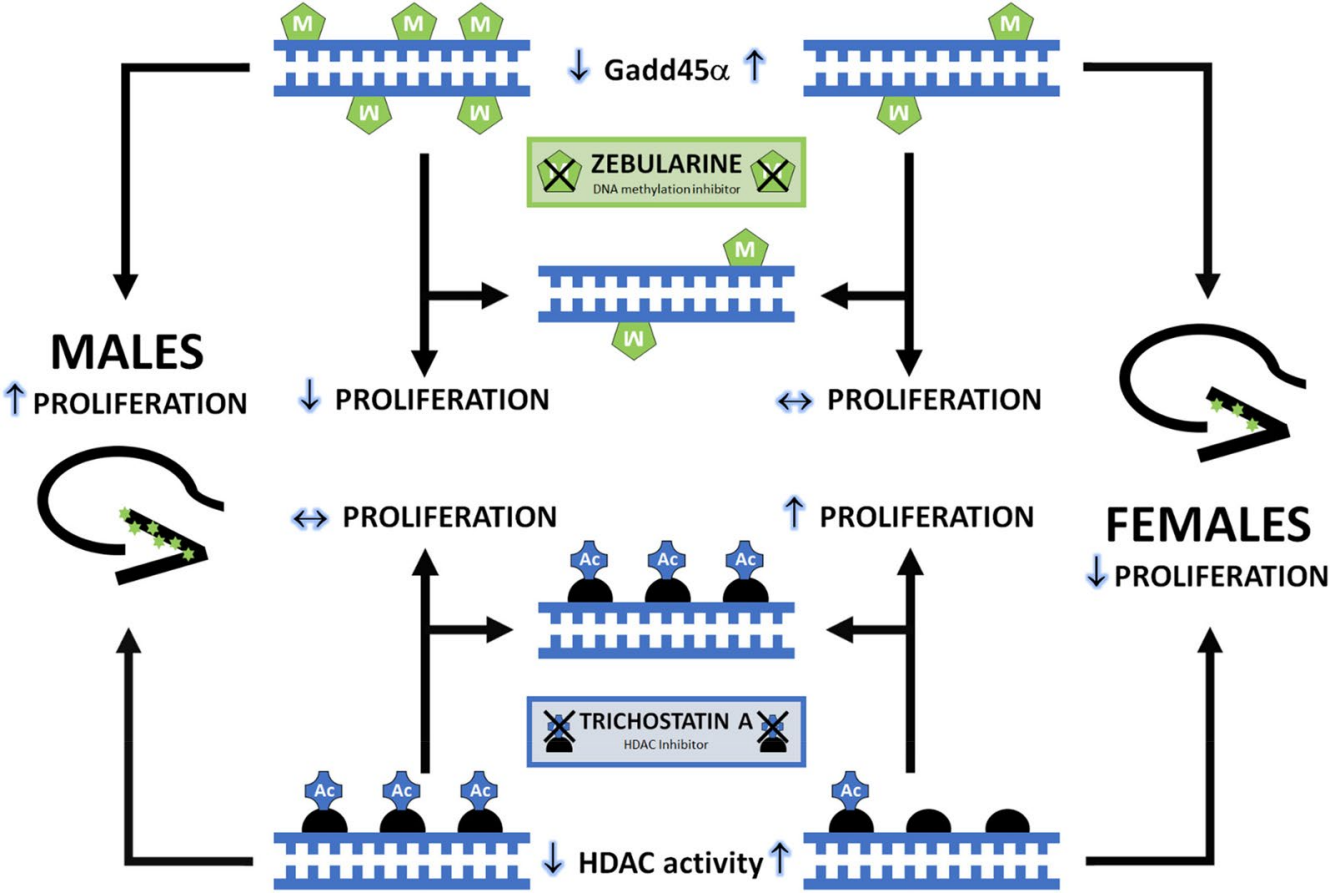
Schematic representation of sexually dimorphic regulation of neonatal DG neurogenesis. In the developing DG, males have more DNA methylation, possibly related to limited demethylation with relatively less expression of Gadd45α, a factor essential for recruitment of base excision repair factors required for demethylation. Treatment of males with the DNA methylation inhibitor, ZEB, decreases proliferation in the DG, but the drug does not alter proliferation in the female DG. HDAC activity is higher in the developing female DG and treatment with the HDAC inhibitor, TSA, increases proliferation only in the female. This suggests that sexually dimorphic epigenetic regulation converges to promote proliferation in the male DG and suppress proliferation in the female DG to establish the observed sex difference in cell genesis

Epigenetics play an important role in modulating the balance between pro- and anti-proliferative factors, with implications for cell genesis. We here provide evidence of divergent epigenetic mechanisms that converge to establish a sex difference in DG cell genesis by modulating DNA methylation in males and histone acetylation in females. Although the overall levels of DNA methylation in the developing DG are very low, the cells in the newborn male DG have significantly more methylated DNA than those within the female DG. Administration of a DNMT inhibitor eliminated this sex difference in DNA methylation by reducing it only in the male DG. Similarly, DNMT inhibition significantly decreased cell proliferation in the DG only in males, such that treated males and females produced similar numbers of new cells. This suggests that elevated methylation in the male DG contributes to greater neurogenesis during early development. By contrast, HDAC activity was higher in female DG compared to male and treatment with an HDAC inhibitor caused a greater proportional inhibition of HDAC activity in the DG of females, thus eliminating the sex difference. Treatment with an HDAC inhibitor also increased cell proliferation in female DG but did not cause any further increase in male DG, indicating that elevated HDAC activity in female DG suppresses neurogenesis. Taken together, these data provide evidence that different epigenetic modifications regulate neonatal cell genesis in opposite directions in males versus females, as graphically presented in Fig. 8. An important limitation to this study is the use of broad-acting pharmacological agents, such as ZEB and TSA, combined with a lack of information regarding in which cells the epigenetic modifications are occurring. It is possible that the cells that are poised to or have recently proliferated are more responsive to epigenetic modifiers, but it is equally possible that signals emanating from neighboring cells are the critical signals regulating the proliferation process.

Higher levels of DNA methylation in the newborn male DG could either be established through enhanced addition of methyl groups or through diminished removal. Sexual differentiation of DNA methylation in other brain regions, including the POA, is achieved through differences in DNMT amount or activity [30, 31]. In the neonatal DG, males did not express higher levels of DNMTs or exhibit greater DNMT activity, suggesting little to no role for enhanced methyl addition. Instead, expression of GADD45α, which recruits base-excision-repair proteins to sites of nucleotide mismatch to induce excision of the deaminated or oxidized 5mC [32, 33], was decreased in the DG of males relative to females. Surprisingly, females expressed higher levels of the maintenance methyltransferase, DNMT1. Genetic deletion of DNMT1 results in significant hypomethylation [34, 35], but overexpression of DNMT1 does not induce hypermethylation [36, 37]. Therefore, it is not surprising that greater DNMT1 expression in the female DG did not manifest in higher global DNA methylation. What purpose if any the elevated DNMT1 mRNA plays in females is unknown.

Levels of enzyme expression do not necessarily translate to enzyme activity, which is ultimately responsible for altering patterns of DNA methylation. No sex difference was identified in DNMT activity in the DG 1 day after birth, when sex differences in neurogenesis are high. The assay utilized to assess DNMT activity quantifies total enzyme activity, summating activity of both de novo and maintenance methyltransferase enzymes. Greater DNMT1 expression in the DG of females complicates the interpretation of DNMT activity, as DNMT1 expression could potentially compensate for less activity of de novo methyltransferases. More specific analysis of individual methyltransferase activities are necessary to rule out contributions of DNMT activity to the sex difference in global DNA methylation, but such assays are not available at this time.

Despite robust differences in HDAC activity in the DG of males and females, the levels of global H3 and H4 histone did not differ. This could be the result of differential targeting of particular genes. Histone-3 lysine-4 trimethylation (H3K4me3) is a histone modification enriched at active promoters near transcription start sites and associated with active transcription. In the bed nucleus of the stria terminalis/preoptic area, the majority of gene loci exhibit similar H3K4me3 enrichment in males and in females, but over 200 of these loci display sex differences in their degree of trimethylation of H3K4 [30]. In females, genes targeted for greater H3K4me3 enrichment are related to synaptic transmission, emotion/affective behavior and learning/memory, whereas higher enrichment of this marker in males modulated genes involved in embryogenesis, development and brain morphology [38]. Despite dynamic sex differences in genes targeted by H3K4me3, there is no significant difference in total H3K4me3 between the sexes [38]. This suggests that global assessment of histone modifications may not always accurately depict the dynamic changes of these marks at specific gene sites. In the current study, enhanced HDAC activity in the DG of neonatal females did not drive sex differences in global histone acetylation, but it is possible that greater deacetylation in females targeted specific genes which were not apparent when using our broad approach.

Cell genesis in the developing brain is a carefully regulated process, controlled by dynamic expression of pro- and anti-proliferative genes, which at times differs between the male and female brain [39], and may be reflective of the differential developmental trajectories of the two sexes, including the hippocampus [40, 41]. Numerous studies confirm a role for BDNF in promotion of proliferation [42]. Males have a higher level of BDNF transcripts in the DG during the first postnatal week [43], which is the same time they make more new cells compared to female littermates. Thus, BDNF was investigated as a possible pro-proliferative gene target silenced by enhanced HDAC activity in females. While the sex difference in BDNF expression was confirmed in the newborn DG, HDAC inhibition did not upregulate BDNF in the female. Targeted suppression of the neurotrophin by greater HDAC activity in the female DG, therefore, could not account for the sex difference in expression. Interestingly, treatment with an HDAC inhibitor decreased BDNF expression in the male DG. If there was greater deacetylation of BDNF itself in the male DG, inhibition of deacetylation by TSA would be predicted to increase expression. That decreased acetylation decreases BDNF expression has been demonstrated in a model of stress [44]. Thus, the fact that we observed that inhibition of deacetylation decreased BDNF expression in the male DG suggests complex epigenetic regulation, whereby a gene or perhaps some other epigenetic regulator that normally suppresses BDNF expression is differentially targeted by deacetylation. Even more surprising, DNMT inhibition reduced expression of BDNF only in the male DG, while expression in the female DG was unchanged, suggesting enhanced methylation promotes BDNF expression. Prevailing dogma dictates that DNA methylation induces transcriptional repression and gene silencing; however, some DNA methylation is permissive and this depends on the location in the genome. Genome-wide sequencing studies reveal that while DNA methylation of CpG islands located in promoter regions within the vicinity of transcriptional start sites repress transcription, methylation within gene bodies positively correlates with greater expression of the associated gene [45–49].

The effects of ZEB treatment on inhibition of DNA methylation, suppression of proliferation and BDNF transcription, were all specific to the male DG, with no effect on any of these end-points in females, suggesting a sex-dependent sensitivity to this drug in the neonatal DG. ZEB is a cytidine analog substrate of DNMTs that can initiate a methylation reaction but then forms a covalent complex with the DNMT when incorporated into DNA [50]. The unresolved bond halts methylation, causing passive loss of DNA methylation, but also compromises the integrity of the DNA, thereby initiating damage signaling and resulting in degradation of the trapped DNMTs. ZEB and related cytidine analogs also act as anti-cancer therapeutics following integration into the DNA of rapidly proliferating cells and inducing apoptosis through a variety of pathways [51, 52]. It is possible the higher rate of proliferation in the DG of males is more permissive to the actions of ZEB than that in females.

Masculinization is mediated by gonadal steroids during the perinatal period, whereas feminization requires no active secretion of ovarian steroids [53]. The absence of hormones to feminize the developing brain has contributed to the belief that the neural anatomy and physiology in the female brain arises by default. Recent research suggests a paradigm shift away from this traditional view of brain sexual differentiation and indicates that feminization of the brain requires active suppression of masculinization. While the overall level of DNA methylation in the developing POA is relatively low, the greater DNA methylation, subsequent to higher DNMT activity, in the developing POA of females silences genes necessary for masculinization [31]. Thus, the female brain is not simply the default developmental pathway. Feminization is instead an active epigenetic process that suppresses masculinization. Our discovery that females have greater HDAC activity in the developing hippocampus further implicates epigenetic suppression as a primary mechanism in females to prevent masculinization throughout the brain. In the female brain, active demasculinization in the absence of feminizing hormones necessitates consideration of mechanisms beyond the dominant hormonal theory of sexual differentiation. All sex differences ultimately stem from the inherent imbalance of genes encoded by the sex chromosomes, but identifying genes guiding sex-specific brain development has been challenging [54]. The X-chromosome is rich in genes associated with epigenetic modification, including two histone demethylases which have paralogs on the Y chromosome [54]. We did not explore the potential for sex differences in histone methylation, as no pharmacologic agent has yet been identified to manipulate this epigenetic modification. This is an important area for future investigation. More intriguingly, however, the sex chromosome composition can also influence sensitivity to gonadal steroids by modulating activity of the estrogen-synthetic enzyme, aromatase, and expression of receptors for estrogen [55, 56]. A similar, but as yet undetected phenomenon of regulation in the developing hippocampus could provide insight into the origins of the sex difference in neurogenesis.

## Data Availability

The data sets used during the current study are available from the corresponding author upon reasonable request.
